# Kismeth: Analyzer of plant methylation states through bisulfite sequencing

**DOI:** 10.1186/1471-2105-9-371

**Published:** 2008-09-11

**Authors:** Eyal Gruntman, Yijun Qi, R Keith Slotkin, Ted Roeder, Robert A Martienssen, Ravi Sachidanandam

**Affiliations:** 1 1 Bungtown Road, Cold Spring Harbor Laboratory, Cold Spring Harbor, NY 11724, USA; 2 National Institute of Biological Sciences, Beijing, No. 7 Science Park Road, Zhongguancun Life Science Park, Beijing, 102206, PR China; 3 Department of Genetics and Genomic Sciences, Mount Sinai School of Medicine, 1425 Madison Avenue, New York, NY 10029, USA

## Abstract

**Background:**

There is great interest in probing the temporal and spatial patterns of cytosine methylation states in genomes of a variety of organisms. It is hoped that this will shed light on the biological roles of DNA methylation in the epigenetic control of gene expression. Bisulfite sequencing refers to the treatment of isolated DNA with sodium bisulfite to convert unmethylated cytosine to uracil, with PCR converting the uracil to thymidine followed by sequencing of the resultant DNA to detect DNA methylation. For the study of DNA methylation, plants provide an excellent model system, since they can tolerate major changes in their DNA methylation patterns and have long been studied for the effects of DNA methylation on transposons and epimutations. However, in contrast to the situation in animals, there aren't many tools that analyze bisulfite data in plants, which can exhibit methylation of cytosines in a variety of sequence contexts (CG, CHG, and CHH).

**Results:**

Kismeth  is a web-based tool for bisulfite sequencing analysis. Kismeth was designed to be used with plants, since it considers potential cytosine methylation in any sequence context (CG, CHG, and CHH). It provides a tool for the design of bisulfite primers as well as several tools for the analysis of the bisulfite sequencing results. Kismeth is not limited to data from plants, as it can be used with data from any species.

**Conclusion:**

Kismeth simplifies bisulfite sequencing analysis. It is the only publicly available tool for the design of bisulfite primers for plants, and one of the few tools for the analysis of methylation patterns in plants. It facilitates analysis at both global and local scales, demonstrated in the examples cited in the text, allowing dissection of the genetic pathways involved in DNA methylation. Kismeth can also be used to study methylation states in different tissues and disease cells compared to a reference sequence.

## Background

DNA methylation involves the conversion of cytosine to 5-methylcytosine, which results from the action of DNA methyltransferases (DNMTs) [1]. DNA methylation occurs in different sequence contexts in different organisms. In *H. sapiens *and other mammals, it is believed that DNA methylation occurs mainly in the cytosines of CG dinucleotides [2].

In plants, DNA methylation is critical for parental imprinting, the regulation of embryogenesis, transposon silencing and for seed viability [3-5]. It has been shown that different pathways are involved in the methylation of cytosines in three different contexts; CG, CHG (C followed by a non-G followed by a G) and CHH (C followed by two non-Gs) [1]. Plants share some of the key elements of the DNA methylation machinery with mammals, but additionally contain plant-specific pathways as well. Plants can tolerate mutations in the DNA methylation pathways, that are embryonic lethal in mammals (e.g. DNMT1), and therefore provide a powerful model system for the study of methylation.

The ability to measure DNA methylation efficiently and accurately is essential for understanding the mechanisms of the processes that lead to DNA methylation. Various techniques have been developed to detect and quantify DNA methylation. Bisulfite sequencing is becoming the gold standard in methylation studies, since it provides both high resolution in sequence and a quantitative measure of DNA methylation at specific loci [6,7].

Bisulfite sequencing involves bisulfite treatment of single stranded DNA that converts unmethylated cytosines (C's) into uracil while methylated C's remain unconverted. After treatment, the region of interest is PCR amplified, and the PCR product is cloned and sequenced. The PCR amplification of the converted C (to uracil) will result in the replacement of uracil with thymine. By comparing the sequence of the bisulfite-treated DNA with that of untreated DNA, the methylation profile is determined: conversion of a C to T indicates non-methylated C's; in contrast, the absence of C to T conversion indicates protection by the methyl moiety of the C and hence methylation. In a standard bisulfite treatment, thus, several sequencing runs/clones are sampled per sequence. This makes the analysis of the data complex.

Most of the extant web-based tools are designed specifically for mammals, and are, therefore, unable to detect methylation outside the CG context. Currently, the only available tool for the analysis of bisulfite converted DNA in plants is CyMATE [8]. Although this tool provides ample analyses, Kismeth provides additional useful features such as the ability to design primers for PCR amplification of bisulfite-treated DNA, analysis of individual sequenced reads and facilitates the bulk analysis of the many sequences associated with bisulfite-treated methylation detection.

## Results and discussion

The C at any particular position may not be completely methylated in any given tissue, which is a measure of the intrinsic variability. In addition, bisulfite treatment can lead to incomplete conversion, which is the extrinsic noise introduced by the act of measurement. Thus, in order to get a measure of the DNA methylation level, a large number of individual clones of PCR products from multiple biological replicates need to be analyzed. Kismeth is one of a few web-based programs that can perform such an analysis, especially for plants.

In this section, we describe Kismeth, then its use in two pilot studies and conclude with a comparison to other tools that can be used to analyze bisulfite sequencing.

### Tool Description

We describe here the two tools included in Kismeth, the analyser of bisulfite sequencing data and the designer of bisulfite sequencing primers.

#### Bisulfite sequencing analyzer

Kismeth requires two fasta-format files, one file containing the reference sequence and the other containing the results of bisulfite sequencing. The reference sequence should be the minimal sequence (not including the PCR primers) between the flanking PCR primers. There are no restrictions on the lengths of sequences other that the limits placed by the sequencing technologies, the software works well for hundreds of sequences, but very large numbers can lead to the website stalling [6,7].

**Figure 1:**
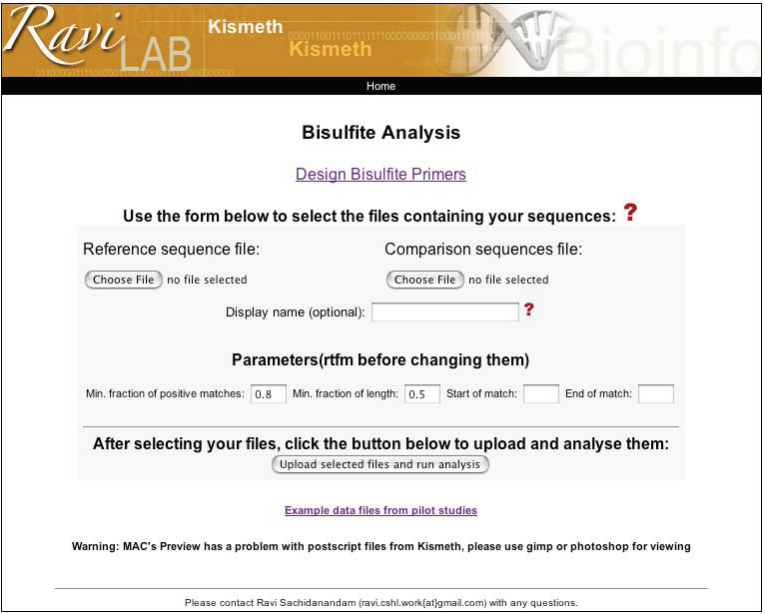
**Front page of the website**. The reference sequence file and a file containing the results of bisulfite sequencing are uploaded in this page. The results are presented as shown in figures 2 and 3. Example datasets described in the text can be downloaded from this page. The parameters used in the program can also be modified through text boxes on this page.

**Figure 2:**
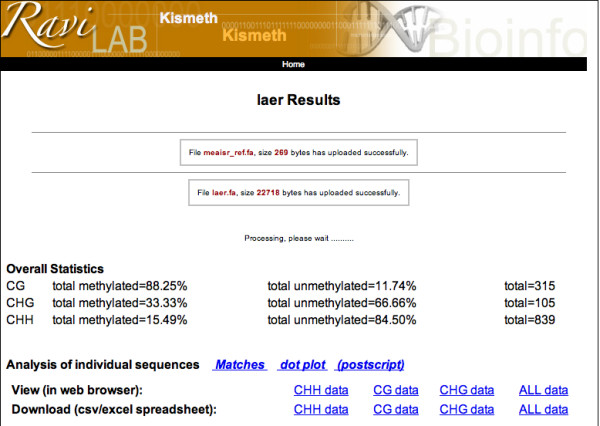
**Synopsis of results (Top)**. This is the top half of the results page. The table shows gross statistics for each type of cytosine (CG, CHG and CHH) for all the sequenced reads. The data for each position can be viewed in the form of tables in webpages (the View links) or as spreadsheets (the download link). The analysis of individual sequences link allows viewing the details alignments (Matches link, shown in figure 4) or as dot-plots showing the cytosines in the sequence (dotplot link, shown in figure 5).

**Figure 3:**
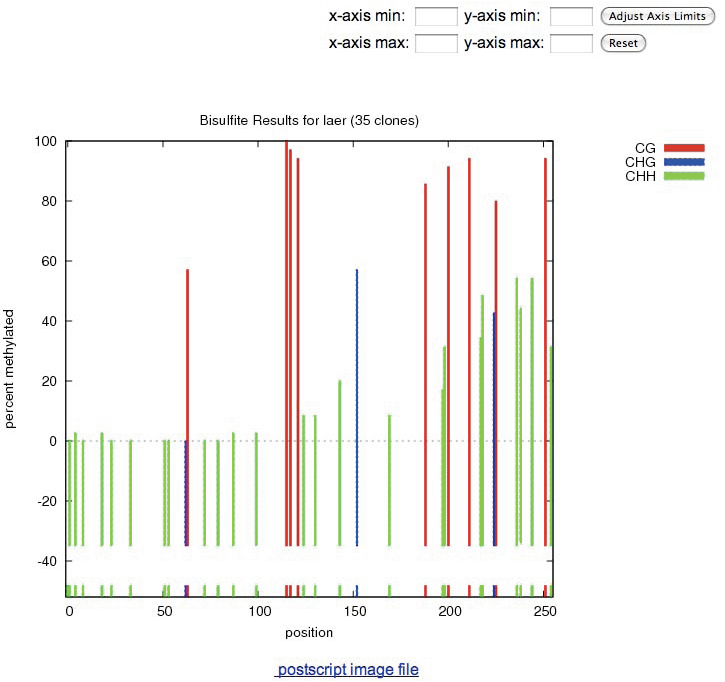
**Synopsis of results (Bottom)**. This is the bottom half of the results page. The graph displays three pieces of information, the location of the various types of C's in the sequence of interest (as colored bars at the bottom of the graph), the number of times each cytosine is sampled in the sequenced reads (as colored bars below the x-axis) and the fraction of times the cytosine at each location is methylated, as colored bars above the x-axis. The colors represent the three types of C's in the sequence, red for CG, blue for CHG and green for CHH. The controls allow zooming into regions of the graph for a closer look.

Both files are uploaded on the front page shown in figure 1. Example files from the pilot study described in the application section are available through a link on the front page of Kismeth. The question mark on the front page provides a manual (or answers to questions) for the tool. Kismeth will perform the analysis and return a synopsis table and graph, shown in figures 2 and 3, summarizing the statistics for the sequence as a whole. The graph shows the fraction of methylation at each cytosine position along the reference sequence, allowing a quick estimate of the rates of methylation in different regions (Figure 3). The data underlying the graph, the methylation states of various kinds of cytosines in the sequence, is also available either for browsing on the web (the *View *links) or as downloadable comma separated value (csv) files (the *download *links) which can be imported into spreadsheet programs.

**Figure 4:**
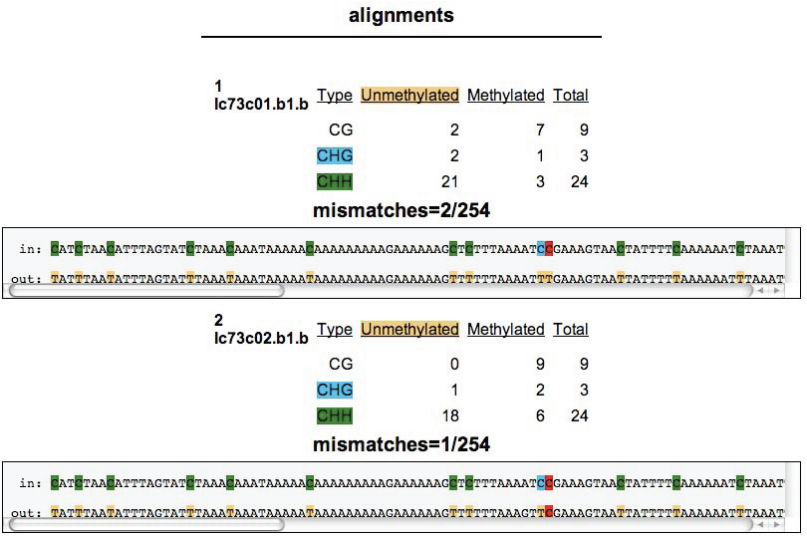
**Detailed view of results for each sequence**. The detailed view of matches highlights various types of cytosines (CG, CHG and CHH) and their fate under bisulfite treatment for each sequenced read. The first line, labeled *in*, of each set is the reference sequence with the cytosines colored to represent the type of cytosine (as in Figure 3), CG (red), CHG (blue) and CHH(green). The second line, labeled *out*, shows the result of bisulfite sequencing with colored T's, *red *for untransformed C's (which are protected by methylation) and *tan *for transformed C's (unmethylated). The mismatches number above each sequence gives the number of mismatches the sequence exhibited (besides the C to T conversions) and the total length of the particular sequence. This provides an estimate for sequence quality.

**Figure 5:**
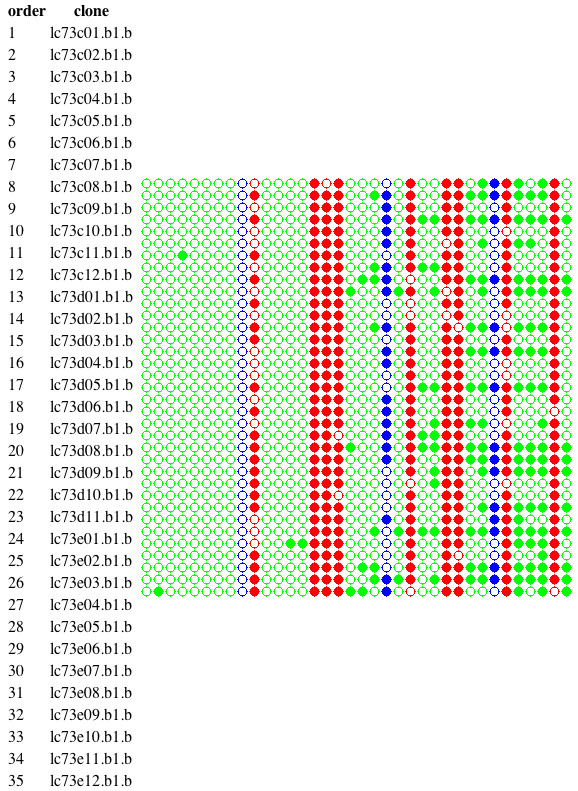
**Dot plot**. The dot plot is a quick way to summarize the fate of the various types of cytosines in each of the individual sequenced reads. Color coded circles are used to represent types (as in Figures 3 and 4), CG (red), CHG (blue) and CHH(green). The circle is filled when the cytosine is methylated. This view is used to detect groups of similar clones within the population that might be masked by averaging (see text). A table of clone names is provided to identify the clones.

In addition, two kinds of detailed reports, on a sequence-by-sequence basis, are accessible through the *Matches *and *dot plot *links on the synopsis page (shown in figure 2). The detailed *matches *view highlights the various kinds of cytosines in the sequence and the result of the bisulfite treatment (figure 4) allowing the user to study individual alignments to estimate the quality of the sequencing effort that can lead to mismatches (besides the C to T conversions). The *dot plot *shows only the cytosines as circles, colored according to the type of cytosine (red for CG, blue for CHG and green for CHH), with filled circles representing methylated cytosines and empty circles representing un-methylated cytosines (figure 5). The program parameters, described in the algorithm section, can be changed on the front page of Kismeth. Kismeth also generates postscript files for various figures, which can be downloaded for use in publications.

#### Bisulfite Primer Design

**Figure 6:**
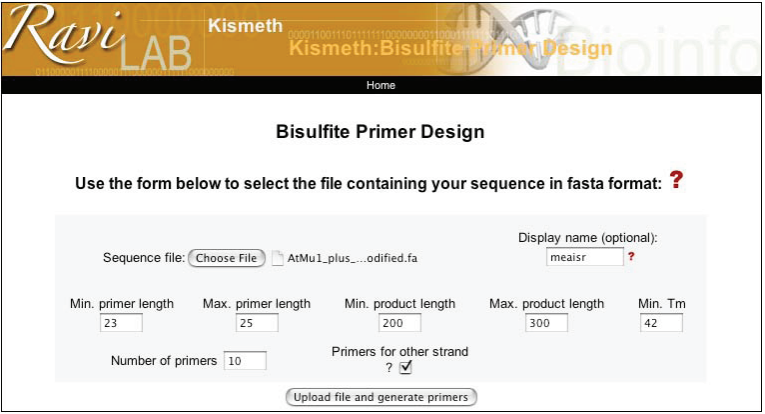
**Design of primers for Bisulfite sequencing**. Through this page the user can upload a reference sequence file, determine the length of the resulting product and the desired Tm, and Kismeth will provide a list of optional primers ranked by their predicted efficiency. The primers are designed taking into account bisulfite limitations, as described in the text. The user can also choose to design primers for the reverse complement of the input sequence, and thus interrogate both DNA strands.

**Figure 7:**
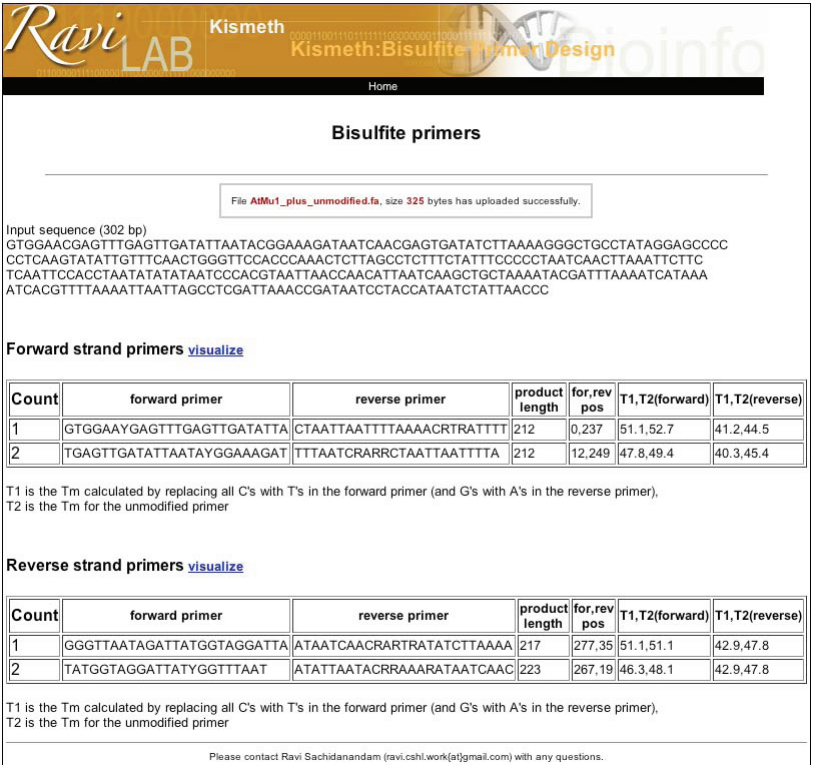
**Primer designs from Kismeth**. The primers pairs are given in the form of a table for each strand, as shown in the figure. We show the results for the *AtMu1 *sequence from the example dataset.

Kismeth also provides the option to design primers for methylation analysis of a particular region. The link for the primer design program on the front page (Figure 1) leads to the primer design front page (Figure 6). Here, the user can upload a reference sequence file, specify the length of the PCR product and the desired Tm (approximate), and Kismeth will provide a list of optional primers based on their predicted efficiency. The user can also choose to design primers for the reverse complement of the input sequence, and thus interrogate both DNA strands. The results are presented as a table (figure 7).

### Application of Kismeth

We used Kismeth to analyze data from two experiments in *Arabidopsis thaliana*. The first study demonstrates the loss of DNA methylation of an *AtMu1 *transposon in a mutant background, leading to epigenetic reactivation [9]. The second study is of the global effect of *ARGONAUTE-4*, which is necessary for CHG DNA methylation in *A. thaliana *[10]. Our aim in these pilot studies is to demonstrate Kismeth's ability to analyze such data in meaningful ways. The biology relevant to these pilot studies is described more extensively in other publications.

#### DNA methylation of an AtMu1 transposable element

Our first pilot study was the use of Kismeth to study methylation data for an *AtMu1 *locus of *A. thaliana *(At4g08680). This transposon is epigenetically silenced by DNA methylation [9]. A *decrease in DNA methylation1 *(*ddm1*) mutant background induces a genome-wide decrease in DNA methylation [11]. The *AtMu1 *locus shows a decrease of DNA methylation in the *ddm1 *mutant background [9].

**Figure 8:**
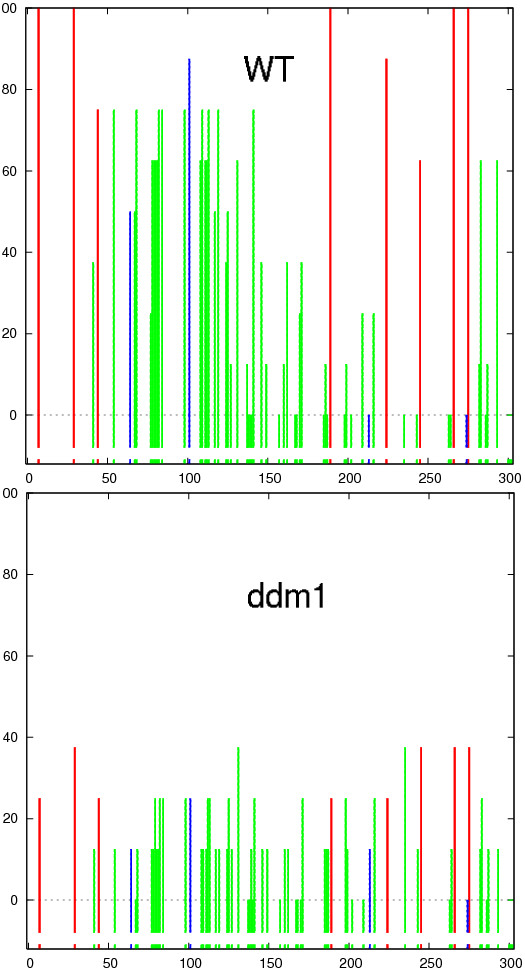
**Graphs for comparison of methylation between WT Col-0 and *ddm1 *mutant over the *AtMu1 *5' terminal inverted repeat (MULE DNA transposon, At4g08680)**. Methylation levels of all cytosines are reduced in the *ddm1 *samples compared to the WT, when averaged across all the clones. Figure conventions the same as in Figure 3.

We generated bisulfite data from wild type *A. thaliana *plants (Columbia-0, Col-0), and the *ddm1 *mutant for the *AtMu1 *5' terminal inverted repeat using PCR primers generated by the primer design tool in Kismeth. As can be seen in Figure 8, changes are apparent in the overall methylation between cytosines of all sequence contexts in the *ddm1 *mutant.

**Figure 9:**
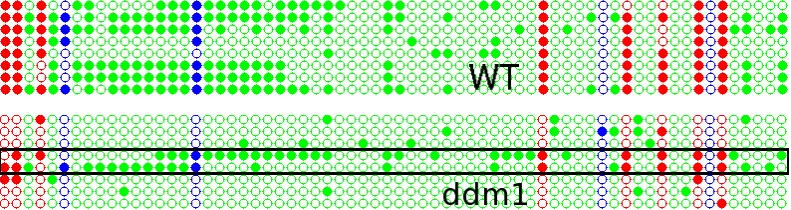
**Dot plot for comparison of methylation between WT Col-0 and *ddm1 *mutant over the *AtMu1 *5' terminal inverted repeat**. Even though the overall methylation is different, two clones appear to retain the WT levels of DNA methylation (boxed region). Figure conventions are the same as in Figure 5.

Using the *Matches *link we can see that even though some of our clones had one or two mismatches, the overall quality was satisfactory. As can be seen in Figure 9, the *dot plot *shows that although there is an overall reduction of methylation in the *ddm1 *mutant, there are two clones in the *ddm1 *background that show wt-like levels of DNA methylation.

The overall decrease in methylation that is evident in Figure 8 can be the result of either a general reduction in methylation levels in each plant or cell, or a complete loss of methylation in some plants/cells and retention of WT levels in others. Figure 9 clearly shows that the latter is correct.

To asses our background non-conversion level we used At2g20610, a gene that is known to be unmethylated in WT. We have provided the data in the example data sets that can be downloaded from the website, it does show that the effect we are seeing is not an experimental artefact.

#### Role of Argonaute-4 in DNA methylation

As a second pilot study, to study global effects of the DNA methylation pathway, we prepared genomic DNA from *A. thaliana *wild type (Landsberg erecta, *La-er*) and an RNAi mutant *ago4-1*. Treated DNA was then used for PCR amplification of MEA-ISR, a repetitive element [12].

In our experiment, *La-er *and *ago4-1 *had 35 and 36 clones sequenced, respectively. These two sets of sequences were then analyzed using Kismeth. The analysis generated by Kismeth was in full agreement with what has been shown previously [10].

**Figure 10:**
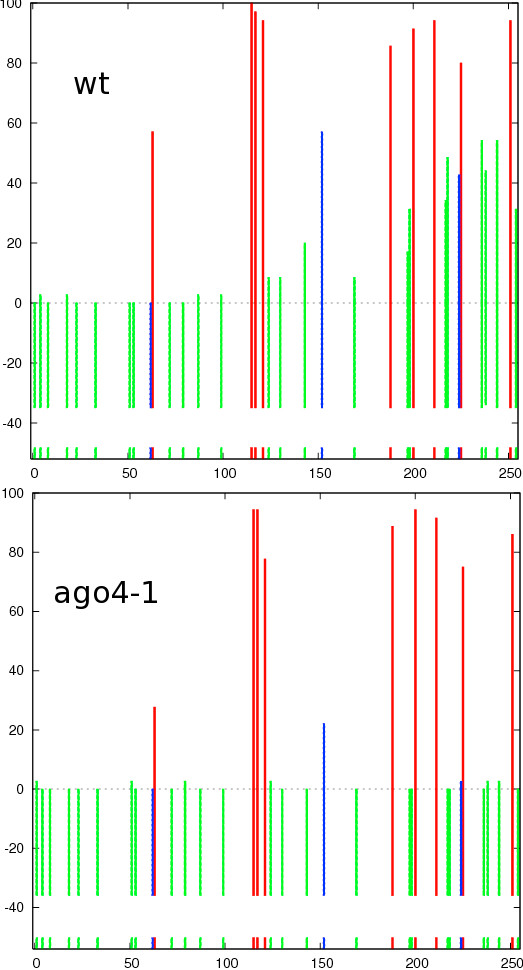
**Comparison of methylation between WT Laer versus *ago4-1 *mutants using Kismeth**. This is a comparison of methylation profiles for WT Laer against *ago4-1 *mutants for MEA-ISR, a repetitive element [12]. The top graph shows the WT while the bottom panel shows the *ago4-1 *mutant. The methylation of the CHG and CHH cytosines is reduced in the *ago4-1 *mutant as compared to the WT, while the CG cytosines are unaffected.

**Figure 11:**
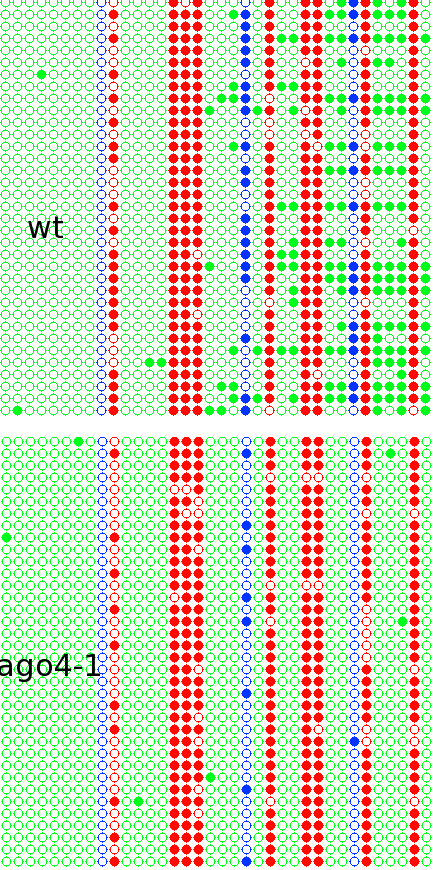
**Dot plot for comparison of methylation between WT Laer versus *ago4-1 *mutants using Kismeth**. This shows a dot plot comparison of methylation profiles for WT Laer against *ago4-1 *mutants. The top dot plot shows the WT while the bottom panel shows the *ago4-1 *mutant. The methylation of the CHG and CHH cytosines is reduced in the *ago4-1 *mutants as compared to the WT, while the CG cytosines are unaffected.

In the wild type plant, high levels of methylated Cs in CG, CHG, and CHH contexts were observed (the example datasets called *Laer *that can be downloaded from the Kismeth website); whereas those in *ago4-1 *(example dataset available on the Kismeth website, labeled *ago4-1*) have a decrease in CHG and CHH methylation, with CG methylation unchanged (Figure 10). The dot plots shown in Figure 11 agree with the observations from Figure 10, that there is a reduction in CHG and CHH methylation by comparing the graphs for the two datasets. Thus, Kismeth allows for a quick evaluation of biologically-relevant, global methylation changes.

### Advantages of Kismeth

There are several programs designed to analyze bisulfite sequencing data, CyMATE [8] is the web-based program that comes closest to Kismeth in terms of functionality and applicability to plant data. We list a few user-friendly features in which Kismeth differs from CyMATE.

• Preparation of Input sequences. Kismeth does not require pre-alignments and the reference sequence must be uploaded separately from the clone sequences. CyMATE takes as input the alignment from ClustalW of the vector-trimmed sequences, with the reference sequence always being the first one.

• Interactive use of the browser. Kismeth presents the results on the browser and alerts users to problems, while CyMATE sends the results via email, some errors, such as incorrect data formats, are indicated via the website.

• Organization of reports and graphs. Kismeth provides graphical output for various aggregate measures as well as raw data files in the form of downloadable, spreadsheet-compatible files whereas CyMATE provides only the dot plot and leaves some of the tables in log files.

• Analysis of individual reads. Kismeth provides a custom viewer (through the *Matches *function) for the study of alignments of the clones against the reference sequence, finding non C/T mismatches and scoring the quality of each read separately, no such facility exists in CyMATE.

• Design of primers. Kismeth is the only tool for bisulfite primer design for plants.

## Conclusion

In animals, DNA methylation is involved in various developmental processes and its dysregulation can cause developmental abnormality and diseases including cancer [13]. In plants, it is critical for parental imprinting, the regulation of embryogenesis, transposon silencing and for seed viability [14]. Detection and measurement of DNA methylation has become an essential component for studying the biology of these processes. Kismeth is a convenient tool for processing data from bisulfite sequencing, the most commonly used method to examine DNA methylation. In all cases, appropriate controls that are not methylated need to also be studied to ensure that there are no systematic biases in the experiments.

Though high-throughput techniques are being developed for the detection of DNA methylation, their validation, for the most part, still relies on traditional bisulfite sequencing [15]. Therefore, tools like Kismeth are still essential for the study of DNA methylation.

## Methods

We describe here the software underlying Kismeth, as well as the algorithms. We also provide details on the experimental methods used in our pilot studies. The use of the tool is described in the Results and Discussion section.

### Kismeth Algorithms and software

The central analysis in Kismeth is the alignment of the bisulfite-treated reads against the reference sequence. This requires that C's in the reference sequence be allowed to align against T's in the bisulfite-treated reads, without a penalty. If a standard, BLASTn-type alignment were used, then regions with a large number of unmethylated C's would not align with the reference sequence, since they would be converted to T's under bisulfite treatment.

One possiblity is to use protein alignment programs, with a custom scoring matrix. We used a *banded *smith-waterman based alignment program, cross_match [16], by modifying the scoring matrix, so that it allows alignment of C's from the reference sequence against T's from the treated sequences.

The sequenced read, as well as its reverse complement, is aligned against the reference sequence, only one of them will align properly, unless the read is of poor quality. Poor alignments, either in terms of the length of match (lengths less than 50 percent of the reference sequence length), or quality of match (less than 80% positive match in the alignment) are not considered for the analysis. These parameters (called *min. fraction of length *and *min. fraction of positive matches*) can be modified on the Kismeth website.

The portion of reference sequence used for analysis can be modified using the *start of match *and *end of match *variables. Sequence ends might have poorer sampling, since the quality of the reads at the ends is usually lower than in the middle, thus care must be taken in inferring position-dependent methylation. The program first identifies the various kinds of C's on the reference sequence (CG, CHG, and CHH). The output of cross_match is parsed by the program and a report is generated, that holds a synopsis for each alignment that is accepted, the alignments, as well as the identities of the various C's in the alignment. This is the central report file that is used to generate various reports and graphs.

The first reports are the gross analysis for the three types of C's in the sequence, which is compiled from the central report file and is shown on the results page. This allows a quick appraisal to see if there is any particular bias in the kinds of C's that get methylated.

A special display program, using the central report file, generates a browseable view of the individual matches. This is available through the *Matches *link on the results page. Various kinds of C's are highlighted using appropriate colors, and the number of mismatches is listed at the top to indicate the overall quality of the match. The start and end of the match on the reference sequence is also shown. This allows access to individual alignments.

Another special display program, again using the central report file, generates a *dot plot*. The dot plot shows only the C's in the reference sequence using appropriately colored circles. Each row represents a read. Open circles are used to represent unmethylated C's while filled circles represent methylated C's. The central report file is also used to generate data files that are used for the plot shown in the figure. The plot is generated using gnuplot, an open source program . We have devised a program that allows zooming into the graph to study details that might not be apparent in the large-scale view of the graph. Separate programs use the same files to generate the table and excel view for the detailed reports on a site-by-site basis.

#### Bisulfite Primer Design

The melting temperature (*Tm*) of the primers is calculated using a crude approximation,

(1) *Tm *= 64.9 + 41 * (number of G's and C's - 16.4)/*N*

where *N *is the length of the primer.

The primers are designed using bisulfite limitations. The software minimizes the C's in the forward primer and the G's in the reverse primer, and does not allow any C in the five bases at the 3' end of the forward primer (or G's in the five bases at the 3' end of the reverse primer).

The forward primers are ranked by the number of C's, with the highest ranking primer being the one with the least number of C's. In addition, the number of C's has to be less than three and not occur in the five bases at the 3' end. If two primers have the same number of C's then the one with the higher *Tm *is ranked higher. For the reverse primers, the number of G's are limited and they are ranked in a similar manner to the forward primers. The C's in the forward primers are replaced with Y (C/T) and the G's in the reverse primers are replace with R (A/G). Pairs of primers are then chosen by picking one from each set, such that they lie within the product length ranges entered by the user.

#### Reagents

For the pilot study on the *AtMu1 *transposable element, the QIAGEN EpiTech bisulfite kit was used according to manufacturer directions. The primer sequences were designed using Kismeth; the forward read primer pair is AATTTTATGGAATGAAGTTATATG and TTCTCATACARTRRCTTCAATTT, while for the other strand, the primer pair is ATAYAGTGGYTTYAATTTGGGTT and RAAAAATATTTRAAAATAACAAAATAAT. The amplified sequences were cloned into a vector using the TOPO TA cloning kit from Invitrogen.

For the pilot study on the effect of Argonaute-4 on DNA methylation, the EZ DNA Methylation-Gold kit was used for bisulfite treatment of genomic DNA according to the manufacturer's instructions (ZYMO Research). We used published primer sequences for MEA-ISR, JP1026 AAAGTGGTTGTAGTTTATGAAAGGTTTTAT and JP1027 CTTAAAAAATTTTCAACTCATTTTTTTTAAAAAA [12]. The PCR products were cloned into pGEM-T easy vector (Promega).

The primers for the control gene (At2g20610) used in the *AtMu1 *study are GTTGYTGATTATATGAAYYGAGATYTT (forward) and TTAATTACAACCATARCCACARTRTTCTC (reverse).

## Availability and requirements

Kismeth is browser-based and publicly accessible through the internet at . There are no restrictions on its use. Kismeth has been tested and found to work on a variety of browsers, Safari, Firefox and Internet Explorer. It has no special requirements except for needing javascript to be allowed to execute in the browser.

## Authors' contributions

YQ suggested the need for a plant-specific tool and helped with the initial design, *ago4-1 *data, testing and writing. EG suggested the need for a dot plot, helped fixed many problems, improved the tool and the writing substantially. RKS edited the manuscript, suggested the primer design program as well as several improvements to the tool in addition to sequencing the *AtMu1 *5' TIR in *ddm1 *and WT. RAM helped with the manuscript and gave the initial impetus for a bisulfite analysis tool. TR designed several aspects of the website. RS designed the tool, created the software and wrote the manuscript. All authors read and approved the final manuscript.

## References

[B1] Chan SW, Henderson IR, Jacobsen SE (2005). Gardening the genome: DNA methylation in Arabidopsis thaliana. Nat Rev Genet.

[B2] Bird A (2002). DNA methylation patterns and epigenetic memory. Genes Dev.

[B3] Jullien P, Kinoshita T, Ohad N, Berger F (2006). Maintenance of DNA methylation during the Arabidopsis life cycle is essential for parental imprinting. Plant Cell.

[B4] Xiao W, Custard K, Brown RC, Lemmon BE, Harada JJ, Goldberg RB, Fischer RL (2006). DNA methylation is critical for Arabidopsis embryogenesis and seed viability. Plant Cell.

[B5] Miura A, Yonebayashi S, Watanabe K, Toyama T, Shimada H, Kakutani T (2001). Mobilization of transposons by a mutation abolishing full DNA methylation in Arabidopsis. Nature.

[B6] Frommer M, McDonald L, Millar D, Collis C, Watt F, Grigg G, Molloy P, Paul C (1992). A genomic sequencing protocol that yields a positive display of 5-methylcytosine residues in individual DNA strands. Proc Natl Acad Sci USA.

[B7] Clark S, Harrison J, Paul C, Frommer M (1994). High sensitivity mapping of methylated cytosines. Nucleic Acids Res.

[B8] Hetzl J, Foerster AM, Raidl G, Mittelsten SO (2007). CyMATE: a new tool for methylation analysis of plant genomic DNA after bisulphite sequencing. Plant J.

[B9] Singer T, Yordan C, Martienssen RA (2001). Robertson's Mutator transposons in A. thaliana are regulated by the chromatin-remodeling gene Decrease in DNA Methylation (DDM1). Genes Dev.

[B10] Zilberman D, Cao X, Jacobsen SE (2003). ARGONAUTE4 control of locus-specific siRNA accumulation and DNA and histone methylation. Science.

[B11] Lippman Z, Gendrel AV, Vaughn MBM, Dedhia N, McCombie WR, Lavine K, Mittal V, May B, Kasschau KD, Carrington JC, Doerge RW, Colot V, Martienssen R (2004). Role of transposable elements in heterochromatin and epigenetic control. Nature.

[B12] Cao X, Jacobsen SE (2002). Locus-specific control of asymmetric and CpNpG methylation by the DRM and CMT3 methyltransferase genes. Proc Natl Acad Sci USA.

[B13] Jones PA, Baylin SB (2002). The fundamental role of epigenetic events in cancer. Nature Reviews Genetics.

[B14] Bender J (2004). DNA methylation and epigenetics. Annu Rev Plant Biol.

[B15] Taylor KH, Kramer RS, Davis JW, Guo J, Duff DJ, Xu D, Caldwell CW, Shi H (2007). Ultradeep Bisulfite Sequencing Analysis of DNA Methylation Patterns in Multiple Gene Promoters by 454 Sequencing. Cancer Research.

[B16] Ewing B, Hillier L, Wendl M, Green P (1998). Basecalling of automated sequencer traces using phred. I. Accuracy assessment. Genome Research.

